# A rare case of mal-positioning of an internal jugular central venous catheter due to an anomalous right upper pulmonary venous return

**DOI:** 10.1177/11297298251319830

**Published:** 2025-02-17

**Authors:** Haidar Hajeh, Ralph Garcia-Pacheco

**Affiliations:** 1 University of California Riverside, Riverside, CA, USA; 2 Kern Medical Center, Bakersfield, CA, USA

**Keywords:** Intensive care, oncology access, interventional radiology, catheters, dialysis access, ultrasonography—Doppler evaluation

## Abstract

Partial anomalous pulmonary venous return is a congenital defect where one or more pulmonary veins drain into the right atrium instead of the left. Most cases are asymptomatic and are discovered incidentally. Anomalous left upper pulmonary venous return is considered the most common type. We present a case of an 84-year-old male who presented to the hospital with altered mentation and suprapubic pain. He was found to be hypotensive and tachycardic and was diagnosed with septic shock of urinary source. He was resuscitated with fluids and antibiotics were started. He continued to be hypotensive and norepinephrine was started. A left internal jugular central venous catheter was inserted with no difficulty and a chest Xray was done for placement confirmation. Xray showed the catheter passing midline to the right hemithorax and pointing toward the right upper lung. A blood gas was drawn from the central catheter and showed pO_2_ of 80 mmHg. A CT scan was performed and showed the catheter coursing into the superior vena cava and pointing toward the right upper lung, wedging into the right upper pulmonary vein that is draining into the superior vena cava. This represents an anomalous right upper pulmonary venous return into the superior vena cava. This would also explain the imaging findings and the unexpected arterial levels of oxygen in the catheter blood gas. The catheter was removed and a femoral central venous access was established.

## Introduction

Partial Anomalous Pulmonary Venous Return (PAPVR) is a congenital anomaly where one or more pulmonary veins drain into the right atrium. It is asymptomatic in most cases but may lead to pulmonary hypertension and right ventricular enlargement in others. It may be associated with other congenital anomalies like atrial septal defects. Surgical management is reserved for cases with impaired functional capacity and right ventricular enlargement.^1^

## Case

**Figure 1.**
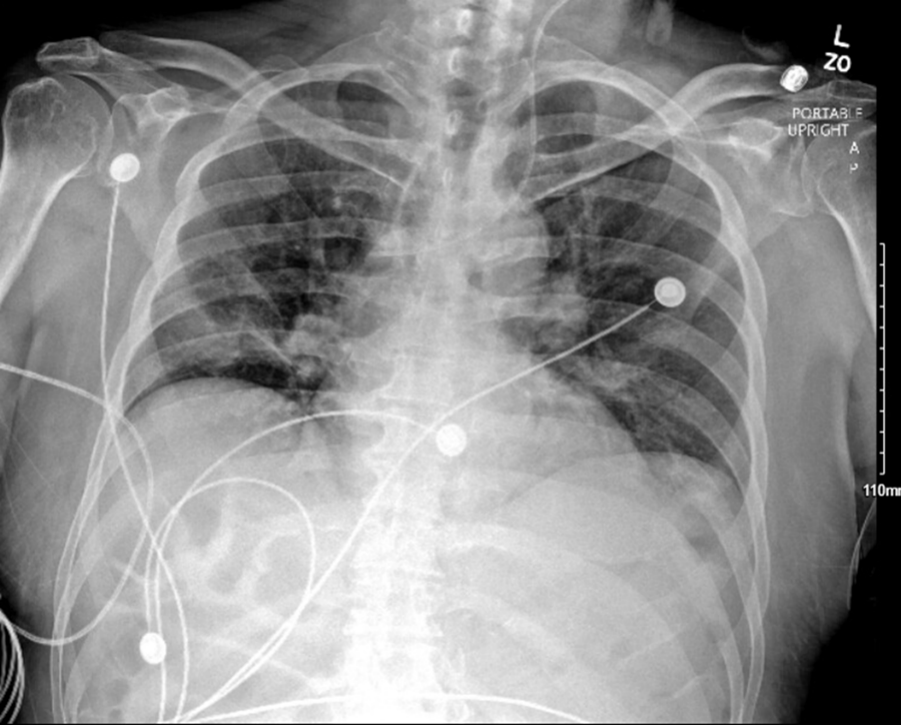
Chest Xray showing the central venous catheter crossing the midline to the right hemithorax and pointing toward the right upper lung.

**Figure 2.**
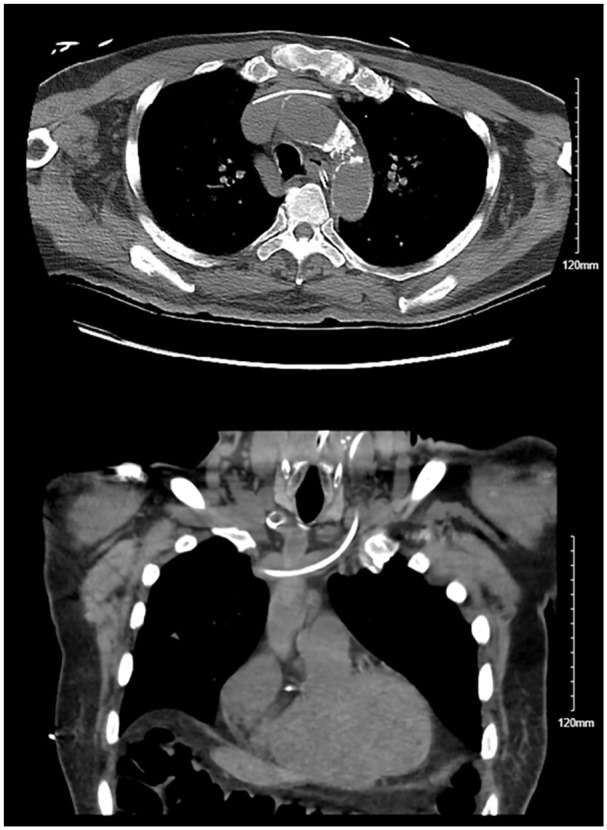
CT scan of the chest with axial (top) and coronal (bottom) images showing the catheter coursing into the Superior Vena Cava (SVC) and wedging into an anomalous right upper pulmonary vein that is draining into the SVC.

An 84-year-old male with a history of coronary artery disease and parkinson’s disease was brought in to the emergency department for altered mentation. Physical examination showed altered mentation with patient oriented to person and year, and suprapubic tenderness. Vitals showed heart rate of 124 bpm and blood pressure of 80/50 mmHg concerning for septic shock of a urinary source. He was resuscitated with a normal saline bolus of 30 mL/kg and broad-spectrum antibiotics were started. Despite resuscitation, patient’s mean arterial blood pressure remained low <65 mmHg necessitating initiation of vasopressors with norepinephrine. A 20-cm triple-lumen central venous catheter was inserted in the left internal jugular vein using the Seldinger technique with ultrasound guidance. Venipuncture of the internal jugular vein produced dark venous-appearing blood and the rest of the procedure was performed with no difficulty. A confirmatory chest Xray (Figure 1) showed the catheter crossing the midline to the right hemithorax and pointing toward the right upper lung instead of terminating at the junction of the superior vena cava and the right atrium. Due to the abnormal appearance on the chest Xray, a blood gas was drawn to confirm the venous nature of the vessel which showed a pO_2_ of 80 mmHg concerning for arterial blood levels. A Computed Tomography (CT) scan of the chest (Figure 2) was performed and showed the catheter coursing into the Superior Vena Cava (SVC) and wedging into an anomalous right upper pulmonary vein that is draining into the SVC. This explains the imaging findings and the arterial oxygen levels in the catheter blood gas.

## Discussion

Pulmonary veins carry oxygenated blood from the lungs to the left atrium. Occasionally, embryological errors result in pulmonary veins draining into the right atrium instead. Total Anomalous Pulmonary Venous Return (TAPVR) occurs when all pulmonary veins drain into the right atrium. TAPVR is usually symptomatic very early after birth with a bad prognosis if early surgical intervention is not performed.^2^ PAPVR on the other hand is usually asymptomatic and clinical presentation depends on the numbers of anomalous veins and concurrent congenital defects among other factors. In its most common form, the left upper pulmonary vein connects to the innominate vein which drains into the SVC. Other forms include a right upper pulmonary vein that drains directly into the SVC as in this patient. Even though no interventions are needed for asymptomatic patients with nonsignificant left-to-right shunting, removal of the central catheter is warranted and a different access should be attempted given the difference in diameter between the right upper pulmonary vein (11.4–12.4 mm)^3^ compared to the larger SVC diameter (15–28 mm)^4^ and the upstream position of the catheter in the anomalous pulmonary vein. In this case, the central catheter was removed with no complications and a femoral access was established. Such anomalies in central venous catheter placement can be avoided by following a systematic method that includes catheter tip location assessment such as the Safe Insertion of Centrally Inserted Central Catheters protocols which recommend techniques such as intracavitary electrocardiography to avoid abnormal catheter tip positioning.^5^ Intracavitary electrocardiography allows the operator to confirm the correct positioning of the catheter tip immediately during the procedure. By connecting a specialized ECG tracer to the wire, we can estimate the location of the catheter/wire tip based on the ECG tracing. An increase in the amplitude of P waves occurs when the tip enters the right atrium necessitating a slight pullback that is accompanied by a depression in the P wave amplitude. This confirms a tip position at the cavoatrial junction.

## Conclusion

Partial anomalous venous return is a rare congenital anomaly that is usually asymptomatic and may be discovered incidentally when placing a central venous catheter. In such cases, removal of the catheter should be considered and a different access site should be attempted.
